# Novel Multiplex Immunoassays for Quantification of IgG against Group B *Streptococcus* Capsular Polysaccharides in Human Sera

**DOI:** 10.1128/mSphere.00273-19

**Published:** 2019-08-07

**Authors:** Giada Buffi, Bruno Galletti, Maria Stella, Daniela Proietti, Evita Balducci, Maria Rosaria Romano, Elena Mori, Monica Fabbrini, Marzia Monica Giuliani, Francesco Berti, Immaculada Margarit

**Affiliations:** a GSK, Siena, Italy; University of Maryland School of Medicine

**Keywords:** GBS, capsular polysaccharides, immunoassay, multiplex, neonatal infection

## Abstract

Group B *Streptococcus* (GBS) infections constitute a major cause of invasive disease during the first three months of life and an unmet medical need that could be addressed by maternal vaccination. The GBS capsular polysaccharides (CPSs) have shown promise as vaccine targets in clinical studies. A highly specific serological assay to quantify maternal and neonatal anti-CPS antibody levels will be instrumental for GBS vaccine licensure. Here, we describe the development and comparison of two novel multiplex immunoassays (MIAs) based on the Luminex technology for the quantification of IgG antibodies recognizing the five most frequent GBS capsular variants (Ia, Ib, II, III, and V) out of the ten types identified. The first assay is based on the use of biotinylated CPSs coupled to streptavidin-derivatized magnetic microspheres (Biotin-CPS MIA), while the second is a sandwich assay with plain CPSs coupled to magnetic microspheres coated with polysaccharide-specific mouse monoclonal antibodies (Sandwich MIA). Both assays showed good specificity, linearity, and precision, although the Biotin-CPS MIA presented higher sensitivity and lower complexity than the Sandwich MIA. A panel of human sera representing a wide range of anti-CPS IgG concentrations was tested in parallel by the two assays, which resulted in comparable titers. Our data support the preservation of antigenic epitopes in the biotinylated polysaccharides and the suitability of the Biotin-CPS MIA for the precise determination of GBS anti-CPS IgG concentrations in human sera.

**IMPORTANCE** Group B streptococcal infections can cause death in neonates up to 3 months of age. Intrapartum antibiotic prophylaxis in GBS-colonized mothers has limited early infections but has no impact after the first week of life. The development of a maternal vaccine to address this unmet medical need has been identified as a priority by the World Health Organization, and the GBS CPSs are considered the best antigen targets. However, to date there are no accepted standardized assays to measure immune responses to the investigational vaccines and for establishment of serocorrelates of protection. Here, we describe the performance of two microsphere-based pentaplex immunoassays for the determination of antibodies recognizing the five most frequent GBS serotypes. Our data confirm that an assay based on biotinylated polysaccharides coupled to streptavidin microspheres would be suitable for the intended purpose.

## Abstract (precis)

Group B streptococcal infections can cause death in neonates up to 3 months of age. Intrapartum antibiotic prophylaxis in GBS-colonized mothers has limited early infections but has no impact after the first week of life. The development of a maternal vaccine to address this unmet medical need has been identified as a priority by the World Health Organization, and the GBS CPSs are considered the best antigen targets. However, to date there are no accepted standardized assays to measure immune responses to the investigational vaccines and for establishment of serocorrelates of protection. Here, we describe the performance of two microsphere-based pentaplex immunoassays for the determination of antibodies recognizing the five most frequent GBS serotypes. Our data confirm that an assay based on biotinylated polysaccharides coupled to streptavidin microspheres would be suitable for the intended purpose.

## INTRODUCTION

The Gram-positive microorganism Streptococcus agalactiae (group B *Streptococcus* [GBS]) is a leading cause of sepsis and meningitis during the first 90 days of life and an important cause of morbidity in the elderly (1, 2). GBS colonizes the genitourinary tract in 11 to 35% of pregnant women and can be transmitted to the neonate during pregnancy, labor, and delivery (3). Intrapartum antibiotic prophylaxis can protect the baby from infection in the first week of life (early-onset disease), but it has some limitations, such as duration of treatment with difficult implementation in resource-poor countries, potential increase of antibiotic resistance, and no effect on late-onset disease (onset between >7 and 90 days after birth) (4, 5). Most GBS are surrounded by a thick layer of capsular polysaccharides (CPSs), of which ten variants/serotypes have been described to date (6). Clinical observational studies have identified an inverse association between the level of GBS serotype-specific anti-CPS IgG antibodies in maternal sera and the risk of GBS invasive disease in neonates, suggesting that GBS vaccination of pregnant women can be a valuable strategy to protect their infants (7).

Monovalent (Ia, Ib, II, III, and V) (8–12) and trivalent (Ia, Ib, and III) vaccines that include CPSs conjugated with tetanus toxoid or with a genetically detoxified mutant of diphtheria toxin (CRM_197_) have been investigated in phase I/II clinical studies (13–18) and have shown good safety and immunogenicity profiles. Functional antibodies elicited by individual CPS variants are not cross-reactive; hence, each single variant is required for protection against the corresponding serotype. A survey of epidemiological studies conducted in different geographical settings led to coverage estimates of up to 86% for the trivalent vaccine, including serotypes Ia, Ib, and III. Considering that five of the ten identified CPS serotypes could cover about 97% of GBS neonatal infection strains, a higher-valency vaccine would be desirable (19).

Clinical studies to assess GBS vaccine efficacy are challenging to design, as they would require a large number of participants; licensure of a GBS vaccine may consequently depend on the establishment of immunological correlates of protection (20, 21). This implies that the development of a quality-ensured standardized serological assay for the precise quantification of antibody levels against the major GBS CPS serotypes is important not only for assessing immune responses in vaccinated subjects but also for establishing antibody levels predictive of neonatal protection from case-control observational studies.

Controversial results were obtained in the past during the development of GBS CPS enzyme-linked immunosorbent assays (ELISAs). The use of unconjugated CPSs as ELISA coating agents resulted in poor assay sensitivity, while conjugated polysaccharides were suspected to expose new epitopes or destroy relevant ones and, thus, alter specific IgG quantification (22–25).

A Luminex-based multiplex assay using CPSs with no or minimal chemical modifications for the simultaneous determination of antibody responses to different GBS serotypes could present several advantages over the former singleton ELISAs. These include epitope conservation, increased sensitivity, a wider dynamic range, and a higher throughput.

**FIG 1:**
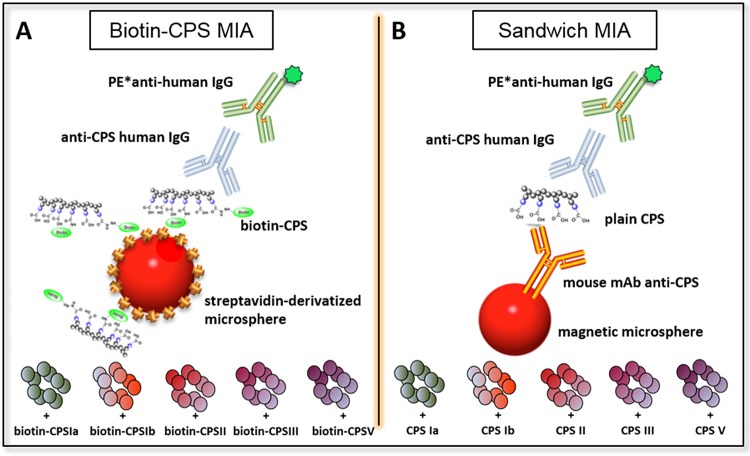
Schematic representation of the pentaplex MIAs using magnetic microspheres containing distinct fluorescent dyes. (A) In Biotin-CPS MIA, biotinylated GBS CPS Ia, Ib, II, III, and V are coupled to streptavidin-derivatized microspheres. (B) In Sandwich MIA, microspheres are coated with anti-GBS CPS Ia, Ib, II, III, and V mouse MAbs to capture serotype-specific plain CPS. PE*, R-phycoerythrin conjugated.

Here, we evaluated the performance of two multiplex immunoassays (MIAs) developed in parallel based on the Luminex technology for quantification of IgG antibodies against CPSs of the most prevalent GBS serotypes, i.e., Ia, Ib, II, III, and V. The first assay is based on the use of biotinylated CPSs coupled to streptavidin-derivatized magnetic microspheres (Biotin-CPS MIA) (Fig. 1A). The second uses five serotype-specific anti-CPS mouse monoclonal antibodies (MAbs) coupled to magnetic microspheres to capture plain CPSs as coating agents (Sandwich MIA) (Fig. 1B).

## RESULTS

### Preparation and analytical characterization of biotinylated CPS Ia, Ib, II, III, and V.

**FIG 2:**
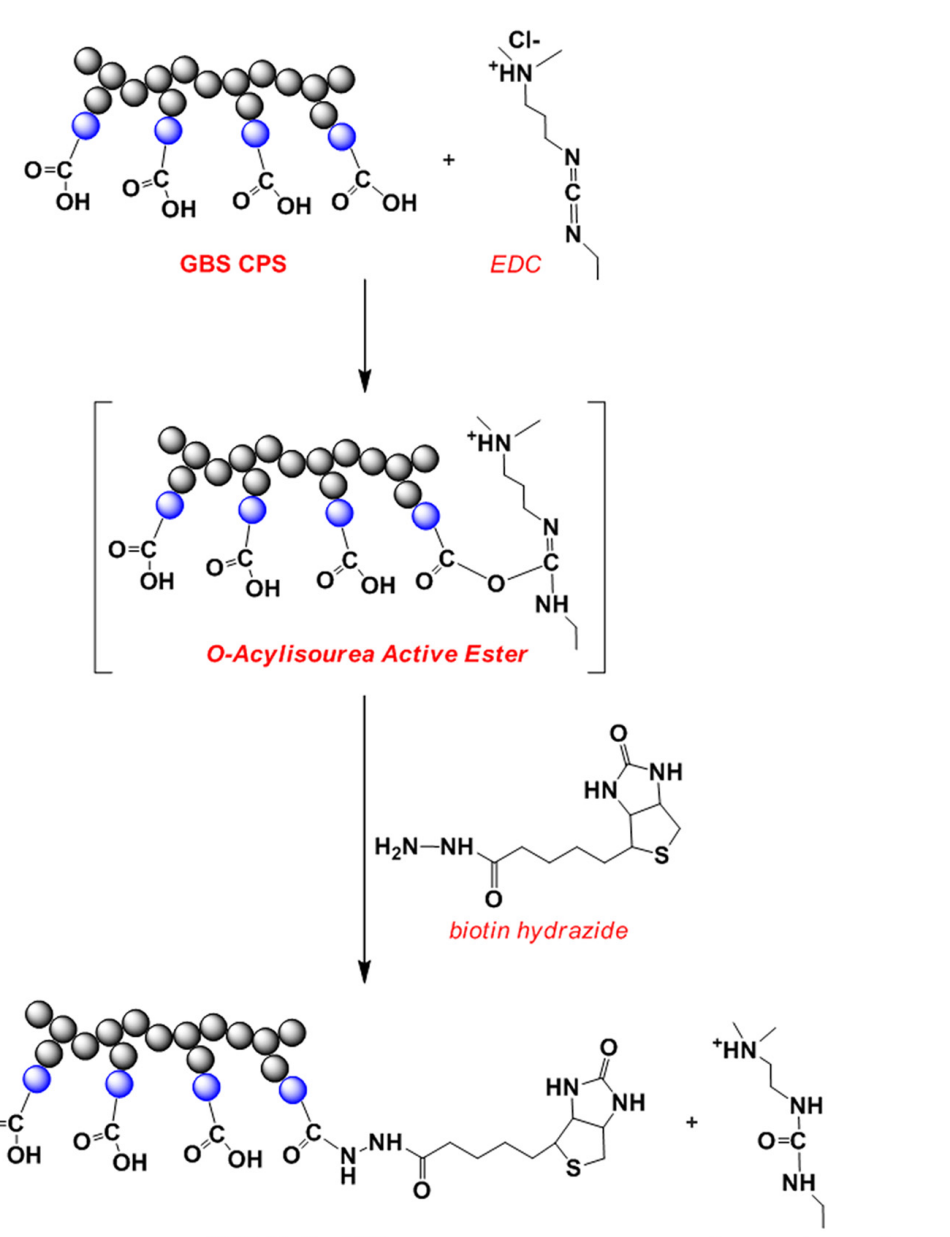
Reaction scheme for biotin-CPS coupling. Blue circles represent the NeuNAc moiety in each CPS RU targeted for covalent linkage to biotin hydrazide by EDC-mediated activation of the carboxylic groups.

For the Biotin-CPS MIA, CPSs were covalently bound to biotin hydrazide by 1-ethyl-3-(3-dimethylaminopropyl)carbodiimide (EDC)-mediated activation of carboxylic groups present on the sialic acid (NeuNAc) moiety of the CPS repeating units (RU) (Fig. 2). The coupling reactions were designed to limit steric constraints and epitope modification in the CPS antigen as well as the amount of unreacted (i.e., nonbiotinylated) CPS. Considering that a single chain of each CPS can contain 100 to 350 RU (corresponding to a molecular weight of approximately 100 to 350 kDa) (26), we assumed that a biotin/CPS molecular ratio around 1% (1 mol of biotin per 100 mol of CPS RU) would result in minimal impact on the epitope and a low residual amount of unreacted CPS.

**FIG 3:**
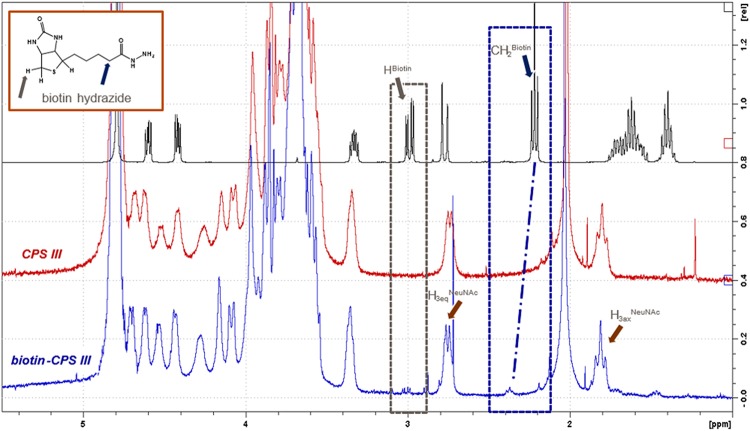
^1^H-NMR spectra of biotin hydrazide reagent, plain CPS III, and biotin-CPS III. Labels at H_3_ NeuNAc and biotin peaks are inserted to facilitate interpretation.

The obtained biotin-CPSs were characterized in terms of total polysaccharide content and NeuNAc retention by high-performance anion-exchange chromatography coupled with pulsed amperometric detection (HPAEC-PAD) (see Table S1 in the supplemental material). The retention of a NeuNAc moiety is considered a key stability indicator of GBS CPSs, because it constitutes the most labile bond of the molecule and is an essential component of the immunodominant protective epitope (27). We assessed CPS identity, structural conformity, and residual free biotin content by ^1^H-nuclear magnetic resonance (NMR), the moles of biotin per CPS chain by QuantTag biotin colorimetric assay, and the moles of unreacted CPS by reverse-phase high-performance liquid chromatography (RP-HPLC) (Table S1). ^1^H-NMR spectra for plain CPS III and biotin-CPS III are shown in Fig. 3. The spectra confirmed the structural identity of CPS III (28) and revealed a downfield shift of the biotin-CH_2_ peak when biotin was bound to CPS III. The relative peak integrals of biotin-CH_2_ and H_3eq_NeuNAc signals evidenced the small amount of inserted biotin molecules per CPS (percentage of biotin, <2% mol biotin/mol CPS RU). Similar results were obtained for CPS Ia, Ib, II, and V (data not shown).

10.1128/mSphere.00273-19.4 TABLE S1 Characteristics of purified Biotin-CPSs. Download Table S1, DOCX file, 0.04 MB. Copyright © 2019 Buffi et al. 2019 Buffi et al. This content is distributed under the terms of the Creative Commons Attribution 4.0 International license.

### Coupling of biotin-CPS to streptavidin microspheres.

To exclude that anti-avidin antibodies that can be present in human sera (29) would be revealed by the Biotin-CPS MIA and interfere with GBS-specific antibody determination, we conducted a preliminary study to select the most appropriate avidin protein for microsphere preparation. First, neutravidin-derivatized magnetic microspheres were used to screen 188 human sera for anti-avidin antibodies. Seventeen of the analyzed sera showed mean fluorescence intensity (MFI) signals above 5,000 at a 1/50 dilution, suggesting the presence of anti-avidin antibodies. These 17 sera were subsequently tested using neutravidin microspheres coupled with biotin-CPS Ia, Ib, and III in the absence or presence of native avidin, neutravidin, or free CPSs as competitors. Three of these sera showed positive signals that were completely inhibited by native avidin or neutravidin but not by free CPSs. The remaining sera were either negative in the absence of competitor or showed signals that could be completely inhibited by CPSs but not by avidin or neutravidin. When the three sera were tested with streptavidin-derivatized magnetic microspheres coupled with biotin-CPS Ia, Ib, and III, the nonspecific signals disappeared. Therefore, we selected streptavidin microspheres for our assay.

**FIG 4:**
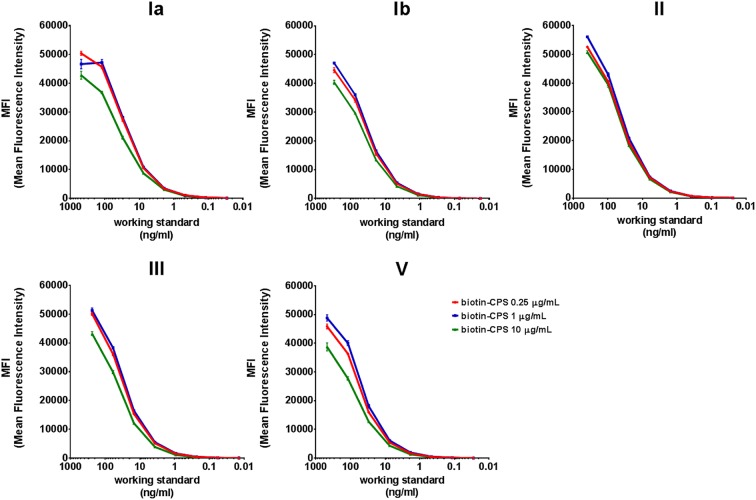
Comparison of MFI signals generated with monoplex Biotin-CPS MIA using a 5-valent human working standard serum and three different biotin-CPS coupling concentrations. Each monoplex curve point is the average from at least three independent values ± standard deviations.

A critical step during the development of the Biotin-CPS MIA was the selection of an appropriate amount of biotinylated CPS that would ensure saturation of the MFI signals when coupled to streptavidin microspheres. Increasing concentrations of biotin-CPS Ia, Ib, II, III, and V (0.25, 1, and 10 μg/ml per 1.25 million microspheres) were tested in triplicate using eight 4-fold serial dilutions of a 5-valent human working standard serum in a monoplex setting. For all serotypes, similar sigmoidal curves were detected for any of the biotin-CPS concentrations, although 0.25 and 1 μg/ml yielded slightly higher MFI values than 10 μg/ml (Fig. 4). Therefore, a concentration of 1 μg/ml was selected for all five CPSs in the final Biotin-CPS MIA.

### Coupling of magnetic microspheres with MAbs and plain CPSs.

The pentaplex Sandwich MIA uses CPS-specific mouse MAbs coupled to magnetic microspheres to capture plain CPS as a coating antigen. The coupling reaction consists of two steps, i.e., covalent conjugation of the microspheres to mouse MAbs recognizing each of the five CPSs (Ia, Ib, II, III, and V), followed by incubation with the respective plain CPS (Fig. 1B).

The concentrations of specific MAbs and free CPSs for microsphere coupling were selected to achieve saturation of MFI signals. For this purpose, a Design of Experiment was used to investigate three MAb concentrations (1, 5, and 25 μg/ml for Ia and 2, 10, and 50 μg/ml for Ib, II, III, and V per 2.5 million microspheres) and three CPS concentrations (0.625, 2.5, and 10 μg/ml per 250,000 microspheres for all serotypes) in a matrix combination by testing three pools of human sera containing high, medium, and low IgG titers against each of the five serotypes. No statistically significant differences in MFI values were observed when using the intermediate and highest concentrations of MAbs for any of the serotypes when tested in combination with the three different CPS concentrations (data not shown). Therefore, intermediate MAb concentrations (5 μg/ml for Ia and 10 μg/ml for Ib, II, III, and V) were selected.

**FIG 5:**
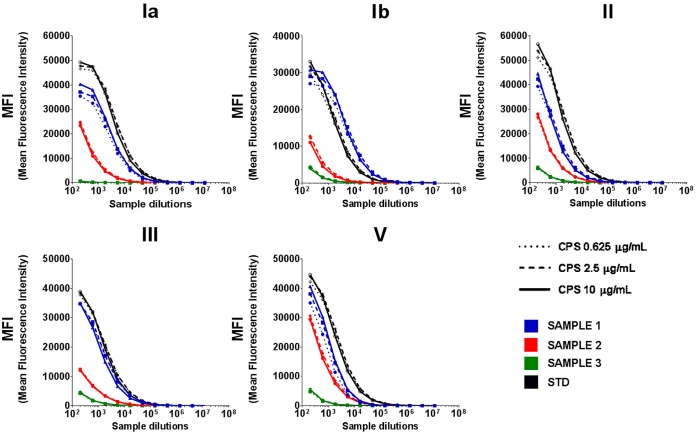
MFI values obtained with the 5-valent human working standard serum (STD) and the pooled human sera with high, medium, and low IgG titers against each of the five serotypes (SAMPLE 1, 2, and 3, respectively) while using 5 μg/ml of anti-Ia MAb and 10 μg/ml of Ib, II, III, and V MAbs and comparing three different concentrations of plain CPS for microsphere coupling.

With the intermediate MAb concentrations, no significant differences in MFI values were detected among the three different CPS concentrations for any of the three pooled samples except one (Fig. 5, sample 1), for which we observed a slightly better signal saturation with the highest CPS concentration for serotypes Ia, Ib, and V. Therefore, the highest concentration of CPS (10 μg/ml) was selected for coating.

Preliminary experiments were conducted to investigate any possible background signals due to recognition of the MAbs used for microsphere coupling by secondary anti-human antibodies. Although a few samples gave some background MFI signals when incubated with microspheres coupled only with the MAbs specific for the five CPS serotypes, the same samples were negative with CPS-MAb coupled microspheres. A negative control consisting of CPS-MAb coupled microspheres incubated with buffer was included in each plate to ensure specificity and to subtract any background signals.

### Optimization of Biotin-CPS and Sandwich MIAs.

For both assays, we investigated the following key parameters in the optimization phase: the reaction buffer, the type of secondary antibody, and the incubation times for primary and secondary antibodies.

Preliminary experiments showed that 0.5% bovine serum albumin (BSA) in the reaction buffer yielded lower background noise on human serum matrix than 0.1% BSA. The type of secondary antibody was selected by comparing R-phycoerythrin anti-human IgGs that reacted either with the Fab or Fc fragments of the human IgG heavy chain. Both types of antibodies provided good signals, but higher sensitivity was achieved when using anti-Fc antibodies. Different incubation times of primary antibodies with coupled microspheres and with secondary antibodies were tested, i.e., 60, 90, and 120 min. We observed that MFI signal saturation required at least 90 min of incubation with the primary antibody and 60 min with the secondary antibody (data not shown).

### Assessment of specificity for Biotin-CPS and Sandwich MIAs.

To exclude the possibility of interference between the microsphere sets, the MFI signals generated from monoplex and pentaplex formats were compared in both immunoassays. The 5-valent human working standard serum was analyzed in duplicate at a starting dilution of 1/200 and ten 3-fold serial dilutions using biotin-CPS and MAb-CPS coupled microspheres. The MFI signals generated in monoplex and pentaplex immunoassays revealed identical standard curves for all combinations of microsphere sets, confirming the feasibility of the multiplex format for both assays (Fig. S1 and S2).

10.1128/mSphere.00273-19.2 FIG S1 MFI curves generated with monoplex and multiplex assays using the Biotin-CPS MIA and serial dilutions of a 5-valent human working standard serum for each of the five serotypes. Each monoplex and pentaplex curve point is the mean ± standard deviation from at least four independent values (duplicates of two independent coupling reactions). Download FIG S1, TIF file, 1.1 MB. Copyright © 2019 Buffi et al. 2019 Buffi et al. This content is distributed under the terms of the Creative Commons Attribution 4.0 International license.

10.1128/mSphere.00273-19.3 FIG S2 MFI curves generated with monoplex and multiplex assays using the Sandwich MIA and serial dilutions of a 5-valent human working standard serum for each of the five serotypes. Each monoplex and pentaplex curve point is the mean ± standard deviation from at least four independent values (duplicates of two independent coupling reactions). Download FIG S2, TIF file, 1.1 MB. Copyright © 2019 Buffi et al. 2019 Buffi et al. This content is distributed under the terms of the Creative Commons Attribution 4.0 International license.

**FIG 6:**
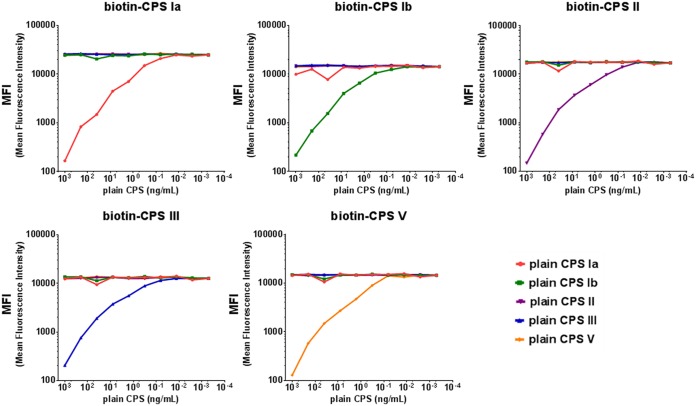
Specificity of the pentaplex Biotin-CPS MIA. MFI values detected with Biotin-CPS Ia, Ib, II, III, and V microspheres after competition of the 5-valent human working standard serum with each of the five plain CPSs at 1 μg/ml and ten 5-fold serial dilutions.

**FIG 7:**
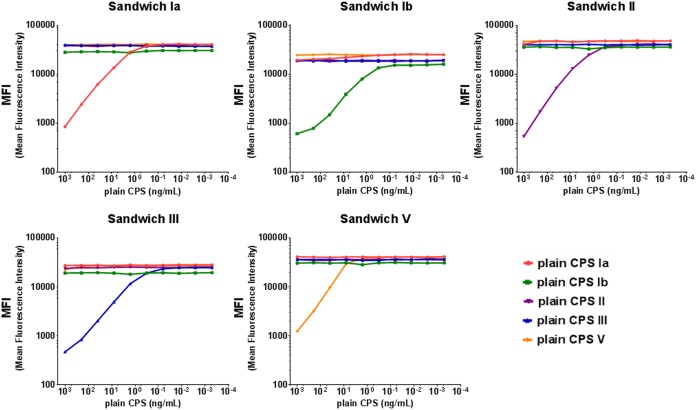
Specificity of the pentaplex Sandwich MIA. MFI values detected with MAb-coupled CPS Ia, Ib, II, III, and V microspheres after competition of the 5-valent human working standard serum with each of the five plain CPSs at 1 μg/ml and ten 5-fold serial dilutions.

Antigen specificity was also assessed by competition experiments. The 5-valent human working standard serum was tested at a fixed dilution in the absence or presence of decreasing concentrations of homologous and heterologous plain CPSs from 1 μg/ml up to ten 5-fold serial dilutions. As shown in Fig. 6 and 7, in both assays addition of homologous antigens resulted in >90% inhibition of CPS-specific MFI signals, while upon addition of heterologous antigens, the inhibition was <10% for all heterologous serotypes.

### Characterization of Biotin-CPS and Sandwich MIAs.

For both the Biotin-CPS and Sandwich MIAs, we established the linearity range for each serotype. Eight test samples were prediluted in a negative matrix and their concentrations measured in duplicate to determine the lower and upper linearity limits (LLLs/ULLs) according to the test procedure. Results are reported in Table 1 and indicated lower LLLs and, therefore, higher sensitivity for the Biotin-CPS MIA than the Sandwich MIA.

**TABLE 1:** Linearity ranges for each serotype with the Biotin-CPS and Sandwich MIAs

Serotype	Linearity limit (ng/ml) for:			
	Biotin-CPS MIA		Sandwich MIA	
	LLL	ULL	LLL	ULL
Ia	121	196,158	753	810,717
Ib	62	486,062	589	687,500
II	239	2,305,419	312	252,828
III	86	652,868	391	5,602,920
V	123	4,690,937	350	2,847,602

The repeatability and reproducibility of the Biotin-CPS and Sandwich MIAs were assessed by determining the level of intra- and interassay variations (repeatability and intermediate precision, respectively). Twelve samples covering a wide range of CPS-specific IgG concentrations were tested in duplicate over 16 runs performed on 8 days by two operators. The coefficient of variation (%CV), describing variability between runs and between plates, indicated an intra- and interassay variability below 15 and 20%, respectively, for both assays (Table 2). The range of intermediate precision across serotypes was 3.7% and 9.2% for the Biotin-CPS and the Sandwich MIAs, respectively.

**TABLE 2:** Coefficient of variation estimated across samples and runs and limits of precision for the Biotin-CPS and Sandwich MIAs^*a*^

Serotype	Biotin-CPS MIA				Sandwich MIA			
	Repeatability (%CV)	Intermediate precision (%CV)	LLP (ng/ml)	ULP (ng/ml)	Repeatability (%CV)	Intermediate precision (%CV)	LLP (ng/ml)	ULP (ng/ml)
Ia	11.0	15.2	233	3,350,688	12.9	14.6	1,774	4,119,822
Ib	11.6	13.2	155	436,309	11.8	12.6	165	499,065
II	14.2	16.9	279	2,200,275	13.7	19.7	348	2,282,025
III	11.1	13.2	293	612,681	12.5	13.2	732	617,918
V	11.7	15.0	168	4,515,231	8.5	10.5	458	4,653,309

a The lower limit of precision (LLP) was defined as the lowest antibody geometric mean concentration for which the %CV of the fitted variance was lower than 25%. The upper limit of precision (ULP) was defined as the highest antibody geometric mean concentration for which the fitted variance corresponded to a %CV of 25%.

Limits of blank (LOBs) were determined based on Clinical and Laboratory Standards Institute guidance EP17-A2 (30) as the 95^th^ percentile of the selected blank measurements determined on at least 46 measurements of negative samples. Limits of detection (LODs) were defined as the lowest theoretical concentration of analyte in a sample that could be consistently detected above the LOB with 95% probability. For each serotype, lower limits of quantification (LLOQs) were set at the highest value among the LLL, the lower limit of precision of the assay (LLP), and the LOD, and upper limits of quantification (ULOQs) were set at the lowest value among the ULL and the upper limit of precision of the assay (ULP).

The obtained estimates for the LOBs, LODs, LLOQs, and ULOQs for the Biotin-CPS and the Sandwich MIAs are reported in Table 3. The LODs and LLOQs were at least 2-fold higher in the Sandwich MIA than the Biotin-CPS MIA for most serotypes, indicating lower sensitivity.

**TABLE 3:** Estimates of LOB, LOD, LLOQ, and ULOQ for the Biotin-CPS and Sandwich MIAs

Serotype	Estimate (ng/ml) for:							
	Biotin-CPS MIA				Sandwich MIA			
	LOB	LOD	LLOQ	ULOQ	LOB	LOD	LLOQ	ULOQ
Ia	73	95	233	196,158	196	263	1,774	810,717
Ib	48	60	155	436,309	127	155	589	499,065
II	82	136	279	2,200,275	215	266	348	252,828
III	52	64	293	612,681	180	231	732	617,918
V	53	62	168	4,515,231	195	220	458	2,847,602

### Comparability of IgG concentrations determined by the two MIAs.

A total of 77 human sera were analyzed to compare IgG concentrations against serotypes Ia, Ib, II, III, and V, obtained by the Biotin-CPS and Sandwich MIAs. The geometric mean titers (GMTs) and %CV for GMT of 3 to 6 test replicates were calculated for each sample. Only samples with %CV for GMT of ≤20% among the replicates were considered for the comparability analysis (71/77 for Ia, 72/77 for Ib, 64/77 for II, 68/77 for III, and 68/77 for V).

The assessment of agreement between the two assays in terms of positive and negative samples (above or below the LLOQ) was conducted by measuring the dissymmetry of the discordance between the number of positive/negative samples (McNemar test). The overall agreement was >90%, and McNemar *P* values were >0.05 for all serotypes except for serotype Ia, for which the agreement was 85.92% and the *P* value was 0.002 (Table 4). This apparent discordance could be explained by the higher LLOQ of the Sandwich MIA for CPS Ia (1,774 ng/ml versus 233 ng/ml for the Biotin-CPS MIA) (Table 3). Indeed, the “discordant” samples showed similarly low IgG values for the two assays (Table S2).

**TABLE 4:** Agreement statistics and McNemar exact *P* value for each serotype

Serotype	Agreement			McNemar (conditional test) exact *P* value
	No./total no. tested	%	95% CI	
Ia	61/71	85.92	75.62; 93.03	0.0020
Ib	67/72	93.06	84.53; 97.71	0.3750
II	62/64	96.88	89.16; 99.62	0.5000
III	67/68	98.53	92.08; 99.96	1.0000
V	63/68	92.65	83.67; 97.57	0.0625

10.1128/mSphere.00273-19.5 TABLE S2 Concentrations (ng/ml) of discordant samples for serotype Ia (positive and negative values for the Biotin-CPS and Sandwich MIAs, respectively). Download Table S2, DOCX file, 0.04 MB. Copyright © 2019 Buffi et al. 2019 Buffi et al. This content is distributed under the terms of the Creative Commons Attribution 4.0 International license.

**FIG 8:**
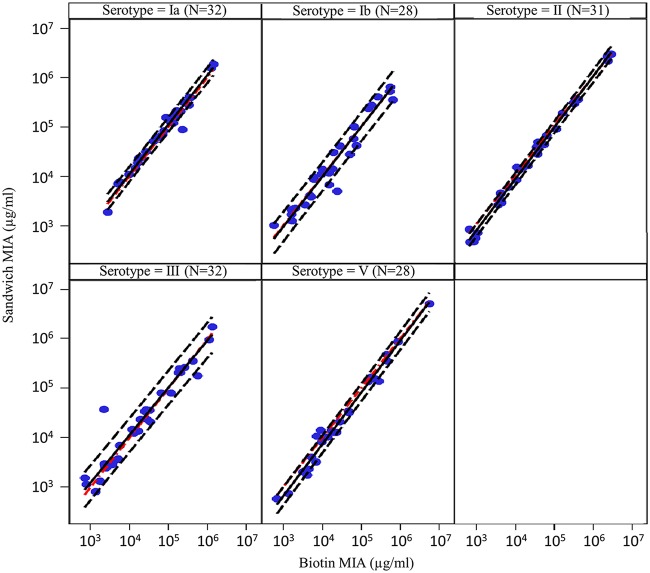
Comparison of measurable serotype-specific IgG concentrations among 77 human samples tested by the Biotin-CPS and Sandwich MIAs using Deming regression on double-positive samples (*N*). The obtained regression line (black continuous), the 80% prediction interval (estimated interval in which a future observation will fall, with 80% probability, based on the observed results; black dashed line) and the reference identity line (red dashed) are shown.

The Deming regression plots of IgG concentrations obtained with the Biotin-CPS and Sandwich MIAs for CPS Ia, Ib, II, III, and V are shown in Fig. 8, along with the 80% prediction interval of future observations based on the obtained results.

All preestablished criteria for assay comparability were met. Indeed, the 95% confidence interval (CI) of the slope was within the preestablished limits of 0.8 to 1.25 for the five serotypes, the 95% CI of the intercept contained the 0 value except for type II (−0.10) and type V (−0.05), and the geometric mean ratio (GMR) of Sandwich MIA GMTs over Biotin-CPS MIA GMTs was within the acceptable range of 0.8 to 1.25 (Table 5). Further, the 80% beta tolerance interval of the plot of the bias was between 0.5 and 2.0.

**TABLE 5:** Intercept, slope of Deming regression, and GMR^*a*^

Serotype	Intercept	95% CI (intercept)	Slope	95% CI (slope)	GMR (95% CI)
Ia	0.03	−0.28; 0.34	1.00	0.94; 1.07	1.11 (1.01; 1.22)
Ib	−0.05	−0.52; 0.41	1.01	0.91; 1.12	0.97 (0.80; 1.18)
II	−0.25	−0.40; −0.10	1.04	1.01; 1.08	0.86 (0.79; 0.93)
III	0.21	−0.26; 0.68	0.96	0.86; 1.07	1.10 (0.88; 1.37)
V	−0.32	−0.59; −0.05	1.05	0.99; 1.11	0.77 (0.69; 0.87)

a Values were obtained by comparing measurable IgG concentrations among 77 human samples tested by Biotin-CPS and Sandwich MIAs.

## DISCUSSION

In this study, we aimed to develop two microsphere multiplex immunoassays based on the Luminex technology for the simultaneous determination of CPS-specific IgG antibodies against GBS Ia, Ib, II, III, and V in human sera. This type of assay allows higher throughput and significant reduction of timing for clinical sample analysis, and it requires lower serum volume than the monovalent ELISAs.

Several approaches that could be compatible with the use of commercially available Luminex microspheres were evaluated for the development of the MIAs. These microspheres contain electrophilic carboxyl groups (-COOH) that are also present in the *N*-acetylglucosamine and NeuNAc moieties of the CPS, while no nucleophilic groups (such as -NH_2_) are available in the CPS molecules for covalent coupling. Therefore, several alternatives were examined. The use of zero-length cross-linking chemistry [e.g., EDC and 4-(4,6-dimethoxy-1,3,5-triazin-2-yl)-4-methylmorpholinium chloride (DMTMM)] for direct coupling or for preparation of aminated microspheres was excluded, as it did not allow physicochemical characterization of the CPS-coupled microspheres due to their solid state (i.e., NMR not feasible). Alternatively, nucleophilic groups could be added to the CPSs by periodate oxidation of the NeuNAc moieties followed by conjugation to -NH_2_ groups of adipic acid dihydrazide using imine chemistry in the presence of NaBH_3_CN (31, 32). However, the same chemistry had been used to obtain the conjugated CPS vaccines (9, 12, 26), and the possibility of creating a new epitope in common with those glycoconjugates could not be excluded. Another possibility was the zero-length cross-linking chemistry with poly-l-lysine to obtain aminated CPSs for microsphere coupling (32, 33). Finally, CPSs could be coupled to the microspheres via a noncovalent high-affinity interaction, i.e., biotin-avidin and antigen-antibody. We reasoned that the last two approaches could better guarantee epitope preservation in the CPS antigen, because the structural integrity of chemically modified CPS (i.e., biotinylated CPS) could be confirmed by analytical testing in solution or by coating microsphere-coupled antibodies with plain CPSs, respectively. Therefore, we decided to develop and compare the performance of the Biotin-CPS and Sandwich MIAs.

For the Biotin-CPS MIA, biotin-derivatized CPSs were prepared by zero-length cross-linking chemistry using biotin hydrazide to achieve an optimal molar ratio of biotin per CPS chain, thereby allowing limited steric constraints and epitope modifications as well as a small amount of unreacted (nonbiotinylated) CPS. This approach offers several advantages. First, an easy purification step allows removal of low-molecular-weight species such as unreacted biotin. Second, the well-defined biotin-CPS molecule can be fully characterized by NMR to determine the structural features at the atomic level. Finally, the one-step coupling reaction of biotin-CPS to streptavidin microspheres is highly efficient and requires a smaller amount of biotin-CPS (microgram scale) than strategies involving chemical coupling (milligram scale).

The multiplex assay using plain CPSs recognized by specific MAbs was evaluated (i.e., Sandwich MIA) as an alternative to biotin-derivatized CPSs. The performance characteristics of the two assays were similar in terms of both specificity and precision. We noted some advantages of the Biotin-CPS MIA over the Sandwich MIA. Preparation of antigen-coupled microspheres for the Sandwich MIA is more complex, as it requires two steps: covalent conjugation of anti-CPS-specific MAbs to microspheres followed by the CPS coupling reaction. In some of the coupling reactions we observed suboptimal antigen loading, resulting in reduced MFI signals that caused assay suitability failure and the need for sample retesting. Further, a large amount of MAbs is needed, and batch-to-batch consistency (purity and stability) could represent an issue in the long term. Moreover, we observed variable results with different lots of secondary antibody with unexpectedly high MFI signals in the blank controls, possibly associated with nonspecific binding of anti-human IgG to the mouse MAbs. Finally, the Biotin-CPS MIA presented higher sensitivity than the Sandwich MIA, with lower LLLs, LLPs, and LODs and consequently lower LLOQs for all the serotypes.

However, the Sandwich MIA was instrumental in demonstrating that the use of CPS conjugated to biotin is a reliable and valid alternative to the use of plain CPSs as coating agents. Indeed, statistical analysis indicated that anti-CPS Ia, Ib, II, III, and V IgG concentrations obtained using the chemically modified biotin-CPS were comparable to those obtained using plain CPSs as coating agents across the intended working range of the assay. Therefore, biotinylation did not expose new cross-reactive epitopes or destroy relevant ones, both of which could have altered the quantitation of anti-CPS antibodies.

Despite previously reported observations of partial cross-reactivity between Ia and Ib serotypes (34), our competition experiments using the 5-valent human working standard serum did not detect any cross-reactivity between these two serotypes. Lack of cross-reactivity was also observed during the comparability study, where seven of the tested sera presented up to 84 μg/ml of anti-Ia IgG but did not show anti-Ib signals and another serum was positive for anti-Ib IgG but did not show anti-Ia signal with either of the two assays. The previously described Ia-Ib cross-reactive antibodies were not functional, and we hypothesize they were directed against a common nonsialylated epitope; it is plausible that high specificity of the CPS-Biotin and Sandwich MIAs is related to the use of fully sialylated CPSs as coating agents, as confirmed by NMR experiments.

GBS assay standardization will be instrumental for the comparison of immunological results from different clinical studies and for the establishment of serological correlates of protection. A global initiative was recently set up with this aim, the GASTON Consortium (7); both purified and biotin-conjugated CPSs along with the Biotin-CPS MIA protocol described in this study were made available to the Consortium.

In conclusion, the developed Biotin-CPS MIA could represent a valuable tool for the precise quantification of human IgGs directed to the main GBS CPSs and, as such, assist the development of a vaccine against this important pathogen.

## MATERIALS AND METHODS

### Preparation and analytical characterization of biotinylated CPSs.

GBS CPSs were purified from serotype Ia, Ib, II, III, and V strains (090, H36B, M781, 18RS21, and 2603, respectively) as described previously (26). CPSs were covalently bound to biotin hydrazide by EDC-mediated activation of carboxylic groups present on the NeuNAc moiety of the RU. The reactions were carried out at pH 5 in 2-(*N*-morpholino)ethanesulfonic acid buffer, using CPS/EDC/biotin hydrazide ratios of 1:1:1 (wt/wt/wt) for serotypes Ia, Ib, III, and V and 1:0.5:0.5 (wt/wt/wt) for serotype II, respectively. The conjugation reactions were initiated by the addition of EDC and biotin hydrazide (solubilized in dimethyl sulfoxide) to the CPS and allowed to react for 16 h while gently stirring at room temperature (RT). The crude mixtures were then purified by tangential flow filtration with a 10-kDa/200-cm^2^ membrane by washing with 1 M sodium chloride (10 dialysis cycles) and then with phosphate-buffered saline (PBS), pH 7.2 (10 dialysis cycles). The purified biotinylated CPSs were characterized in terms of total saccharide content and free NeuNAc content by HPAEC-PAD (27), unreacted CPS by RP-HPLC, structure identity and conformity, residual free biotin content, and NeuNAc retention by ^1^H-NMR, and the biotin/CPS molar ratio was estimated by QuantTag colorimetric assay. These analytical methods are described in detail in Text S1 in the supplemental material.

10.1128/mSphere.00273-19.6 TEXT S1 Analytical methods for characterization of Biotin-CPSs. Download Text S1, DOCX file, 0.04 MB. Copyright © 2019 Buffi et al. 2019 Buffi et al. This content is distributed under the terms of the Creative Commons Attribution 4.0 International license.

### Coupling of biotinylated CPS to the streptavidin microspheres.

Each serotype-specific biotin-CPS was coupled to high-capacity streptavidin-derivatized magnetic microspheres (Radix Biosolutions, USA). Following equilibration at RT, 1.25 million microspheres were transferred to LoBind tubes (Eppendorf) and placed into a magnetic separator for 2 min in the dark, and the supernatant was removed. Microspheres were washed twice with PBS containing 0.05% Tween 20 (Calbiochem). After the second wash, biotin-CPS was added to the microspheres at a final concentration of 1 μg/ml in PBS, 0.05% Tween 20, 0.5% BSA (Sigma-Aldrich). The biotin-CPS–microspheres mixture was incubated for 1 h with end-over-end rotation at RT in the dark and washed twice with PBS, 0.05% Tween 20. Coupled microspheres were suspended in 500 μl of PBS, 0.05% Tween 20, 0.5% BSA and stored at 4°C.

### Protocol for antibody measurement by uncoupled neutravidin microspheres or by Biotin-CPS MIA using neutravidin microspheres.

Neutravidin MagPlex microspheres (Radix Biosolutions) were used uncoupled or coupled to biotin-CPS Ia, Ib, II, III, and V (1.25 million neutravidin microspheres per 1 μg/ml biotin-CPS) as described above for streptavidin microspheres. Native avidin and neutravidin were obtained from Thermo Fisher Scientific.

### Coupling of MAbs and free CPSs to the magnetic microspheres.

Mouse MAbs specific for CPS Ia, Ib, II, III, or V were generated by Areta International (Varese, Italy) using standard protocols. The selected MAbs were purified by protein G affinity chromatography. Each purified anti-CPS MAb (Ia 5 μg/ml, Ib, II, III, and V 10 μg/ml) was coupled to 2.5 million carboxylated MagPlex magnetic microspheres (Luminex Corporation) by following the Luminex protein coupling method (Luminex Corporation). Coupled microspheres were stored at 4°C in PBS, 0.05% Tween 20, 0.5% BSA. Each specific plain CPS, at a final concentration of 10 μg/ml, was incubated with the corresponding MAb-coupled microspheres (250,000) for 1 h with end-over-end rotation at RT in the dark. After incubation, microspheres were washed twice with PBS, 0.05% Tween 20 and stored in PBS, 0.05% Tween 20, 0.5% BSA at 4°C.

### Human sera.

A 5-valent human working standard serum was prepared by pooling 24 hyperimmune human sera from GBS invasive disease convalescent adults (35). The working standard was calibrated against weighed monovalent Ia, Ib, II, III, and V standard human reference sera (10). The working standard contained 182.7 μg/ml anti-Ia, 117.2 μg/ml anti-Ib, 201.3 μg/ml anti-II, 111.9 μg/ml anti-III, and 269.1 μg/ml anti-V IgG. A control serum with CPS-specific IgG between 2 and 10 μg/ml was prepared by pooling human sera from GBS invasive disease convalescent adults (35) for each of the five serotypes and was titrated against the 5-valent human working standard serum.

Out of the 188 human sera used for the selection of the microspheres for development of the Biotin-CPS MIA, 92 were commercial human donor sera (3H Biomedical, Sweden), and 96 were samples from a trivalent GBS vaccine clinical study (14).

Out of the 77 human sera selected for the assay comparability study, 39 were collected in a prospective study from GBS invasive disease convalescent adults (35), 29 were commercial human donor sera (3H Biomedical, Sweden), and nine were from a trivalent GBS vaccine clinical study (18). The selection was based on a preliminary analysis of anti-CPS Ia, Ib, II, III, and V IgG concentrations by Biotin-CPS MIA to reach uniform coverage across the assay working range.

The prospective study was approved by institutional review boards, and informed consents were obtained from eligible patients (35). The vaccine trials were conducted in accordance with Good Clinical Practice guidelines and included informed consent (14, 18).

### Protocol for antibody quantification by Biotin-CPS and Sandwich MIAs.

The same protocol was applied for both the Biotin-CPS and the Sandwich MIAs. Eleven 3-fold serial dilutions of the 5-valent human working standard serum, control serum, or test samples were prepared in PBS, pH 7.2, 0.05% Tween 20, 0.5% BSA. Each serum dilution (50 μl) was mixed with an equal volume of conjugated microspheres (3,000 microspheres/region/well) in a 96-well Greiner plate (Millipore Corporation) and incubated for 90 min at RT in the dark on a plate shaker at 600 rpm. Working standard and control sera were included on each plate. Blanks consisting of biotin-CPS-streptavidin microspheres (Biotin-CPS MIA) and CPS-MAb-MagPlex microspheres (Sandwich MIA) were included in each plate (8 wells). These coupled microspheres were incubated with assay buffer (no serum sample) to subtract background due to incubation with the secondary antibody. After incubation, the microspheres were washed three times with 200 μl PBS. Each well was loaded with 50 μl of 2.5 μg/ml R-phycoerythrin AffiniPure goat anti-human IgG, Fcγ fragment specific (Jackson Immunoresearch), in PBS, pH 7.2, 0.05% Tween 20, 0.5% BSA and incubated for 60 min with continuous shaking. After washing, microspheres were suspended in 100 μl PBS and shaken before the analysis with FlexMAP3D. Data were acquired in real time by Xponent Software (Luminex Corporation).

### Statistical analysis. (i) Fitting of the standard curve and acceptance criteria.

Bio-Plex Manager Software 6.1 (Bio-Rad) was used to fit the model of the standard curve. The MFI signals obtained from eleven 3-fold serial dilutions of the 5-valent human working standard serum were adjusted to blanks and modelled on a logarithmic scale as a function of the theoretical concentration. The resulting dose-response curve displayed a sigmoidal shape and was fitted through the 5-parameter logistic fitting algorithm. The lower and upper limits of the interpolation range were set for each individual standard curve at the MFI values corresponding to the lower and upper limits of standard curve accuracy (LLSCA and ULSCA). These limits were derived from the analysis of 82 standard curves obtained by two operators using 41 independent plates, as the nominal concentrations of 8 out of 11 serial dilution points (from 1/1,800 to 1/3,936,600 for the Biotin-CPS MIA and from 1/600 to 1/1,312,200 for the Sandwich MIA) that presented a 90% prediction interval of relative error between −25% and 25%. For each analyte, the MFI value was converted to micrograms per milliliter by interpolation in the 5-parameter logistic working standard curve (log–log) for every microsphere region/standard. The specific IgG titers were expressed as the GMT of at least three back-calculated concentrations with recovery of 75 to 125% with respect to the median concentration and with interdilutional CV of ≤25%.

**(ii) Repeatability and reproducibility.** A random linear model was applied for the assessment of repeatability and intermediate precision (intra- and interassay variability) to estimate the total variance while including the log_10_-transformed titer as the response and the run as a random factor. A run was defined as the combination of day and operator. The sample was included as a random factor to account for possible interaction between different average titers of each sample and run. This approach is similar to the one described in USP 1033, *Biological assay validation* (36). The random-model analysis of variance was applied simultaneously for all samples as well as for each single sample. The evaluation of the results was performed using PROC MIXED in SAS Enterprise Guide 4.3. On the basis of the results of the MIXED procedure, the total variability was calculated as the sum of the contribution of the intra-assay variability (residual error) and the interassay variability (sum of run and run-by-sample variance components).

**(iii) Linearity.** The analytical range of linearity for each test sample was determined using the dose proportionality approach. The dose proportionality is tested assuming that the logarithm of the measured concentrations is linearly related to the logarithm of the dilutions, log_10_(concentration) = α + β × log_10_(dilution), where β, the slope, measures the proportionality between the dilution and the measured concentration. When β = −1, the dose proportionality is ensured. The range over which dose proportionality is demonstrated was obtained by a recursive search. The first range where the 90% CI for β satisfied the acceptance interval was considered the linearity range.

**(iv) Limits of blank.** LOBs were determined based on the Clinical and Laboratory Standards Institute guidance EP17-A2 as the 95^th^ percentile of the selected blank measurements (30). They were determined on 72, 89, 46, 84, and 83 measurements of negative samples (Text S2) for types Ia, Ib, II, III, and V, respectively, for the Biotin-CPS MIA and on 89, 66, 78, 78, and 135 measurements of negative samples for types Ia, Ib, II, III, and V, respectively, for the Sandwich MIA.

10.1128/mSphere.00273-19.7 TEXT S2 Method for determination of limits of blank. Download Text S2, DOCX file, 0.04 MB. Copyright © 2019 Buffi et al. 2019 Buffi et al. This content is distributed under the terms of the Creative Commons Attribution 4.0 International license.

**(v) Comparability study between the two assays.** Comparability of the data obtained by the two assays across the selected panel of sera was assessed by a qualitative analysis of discordant samples (sera with titers above or below LLOQ) using the McNemar test and by a quantitative analysis comparing paired results from positive samples using a Deming regression. The Deming regression was performed after log_10_ transformation of double-positive data (titers above LLOQ for both assays). The variability of the errors was assumed to be equal in both data sets. The slope and the intercept of the regression line and their corresponding 95% CI were reported. The complete identity line corresponds to a slope equal to 1. The following preestablished acceptance criteria were used as indicators of comparability between the data obtained by the two assays: (i) IgG GMTs estimated from at least three valid tests; (ii) agreement (percentage of positive/positive plus negative/negative) of ≥90%; (iii) McNemar *P* value of ≥0.05; (iv) GMR of 0.8 to 1.25; (vi) 95% CI of Deming regression slope of 0.8 to 1.25; (vii) 95% CI of Deming regression intercept containing 0; and (viii) 80% beta tolerance interval of the plot of the bias of 0.5 to 2.0.
