# Supplementary material for: Novel Multiplex Immunoassays for Quantification of IgG against Group B Streptococcus Capsular Polysaccharides in Human Sera
Source: mSphere. 2019 Aug 7;4(4):e00273-19. doi: 10.1128/mSphere.00273-19 (PMC6686225; doi:10.1128/mSphere.00273-19)
Supplement: TABLE S1 [file mSphere.00273-19-st001.docx]

| Serotype | % Biotin  (Qtag colorimetric assay) | Total saccharide, µg/mL  (HPAEC-PAD assay) | % CPS not biotinylated  (RP-HPLC) |
| --- | --- | --- | --- |
| biotin-CPS Ia | 0.6 | 2,182 | <1.5 |
| biotin-CPS Ib | 1.8 | 1,914 | 3.8 |
| biotin-CPS II | 1.1 | 1,940 | <1.8 |
| biotin-CPS III | 0.6 | 2,112 | 4.1 |
| biotin-CPS V | 0.8 | 1,410 | 11.3 |
