# Supplementary figures and images for: Novel Multiplex Immunoassays for Quantification of IgG against Group B Streptococcus Capsular Polysaccharides in Human Sera
Source: mSphere. 2019 Aug 7;4(4):e00273-19. doi: 10.1128/mSphere.00273-19 (PMC6686225; doi:10.1128/mSphere.00273-19)

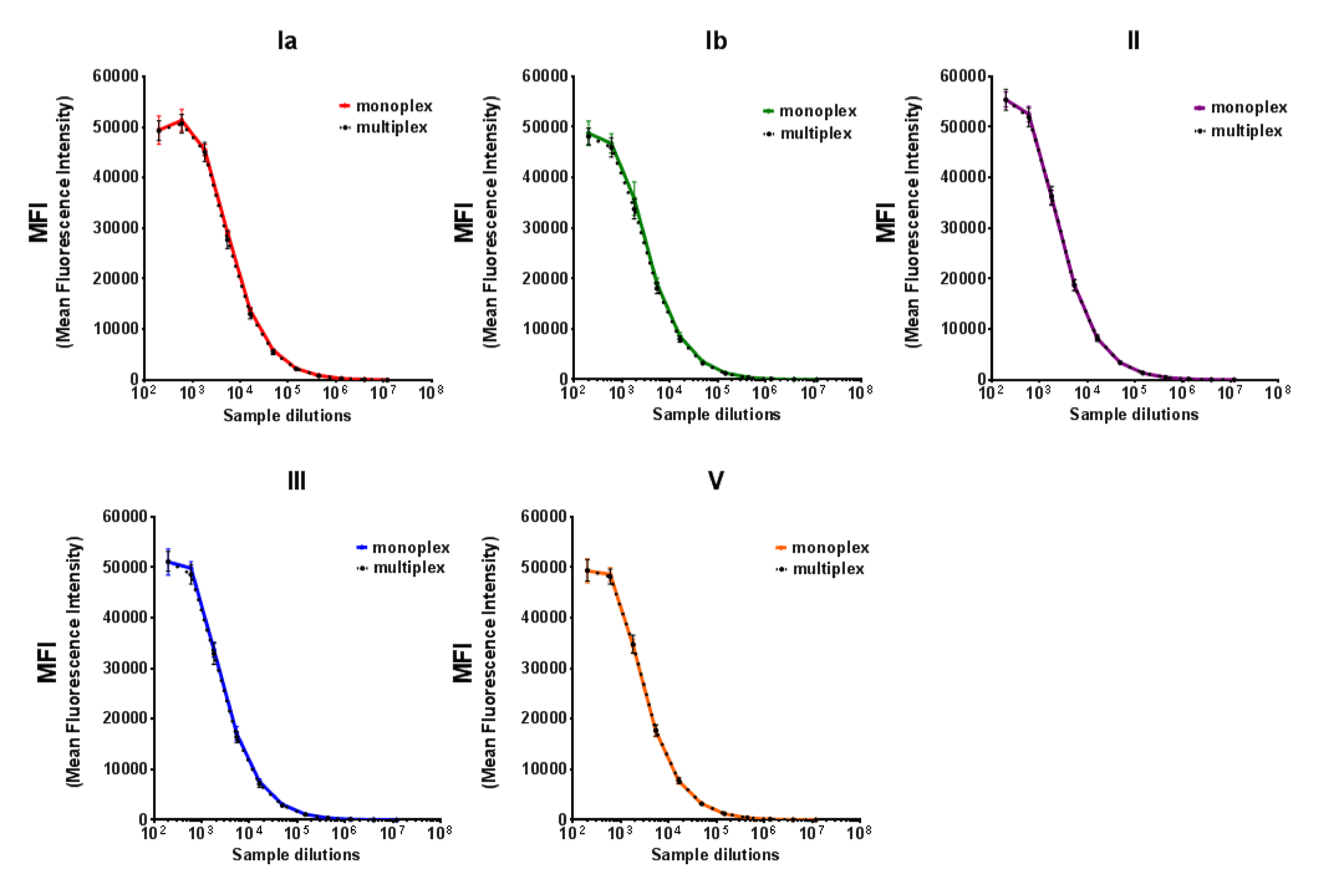

Supplement: FIG S1 [file mSphere.00273-19-sf001.tif]

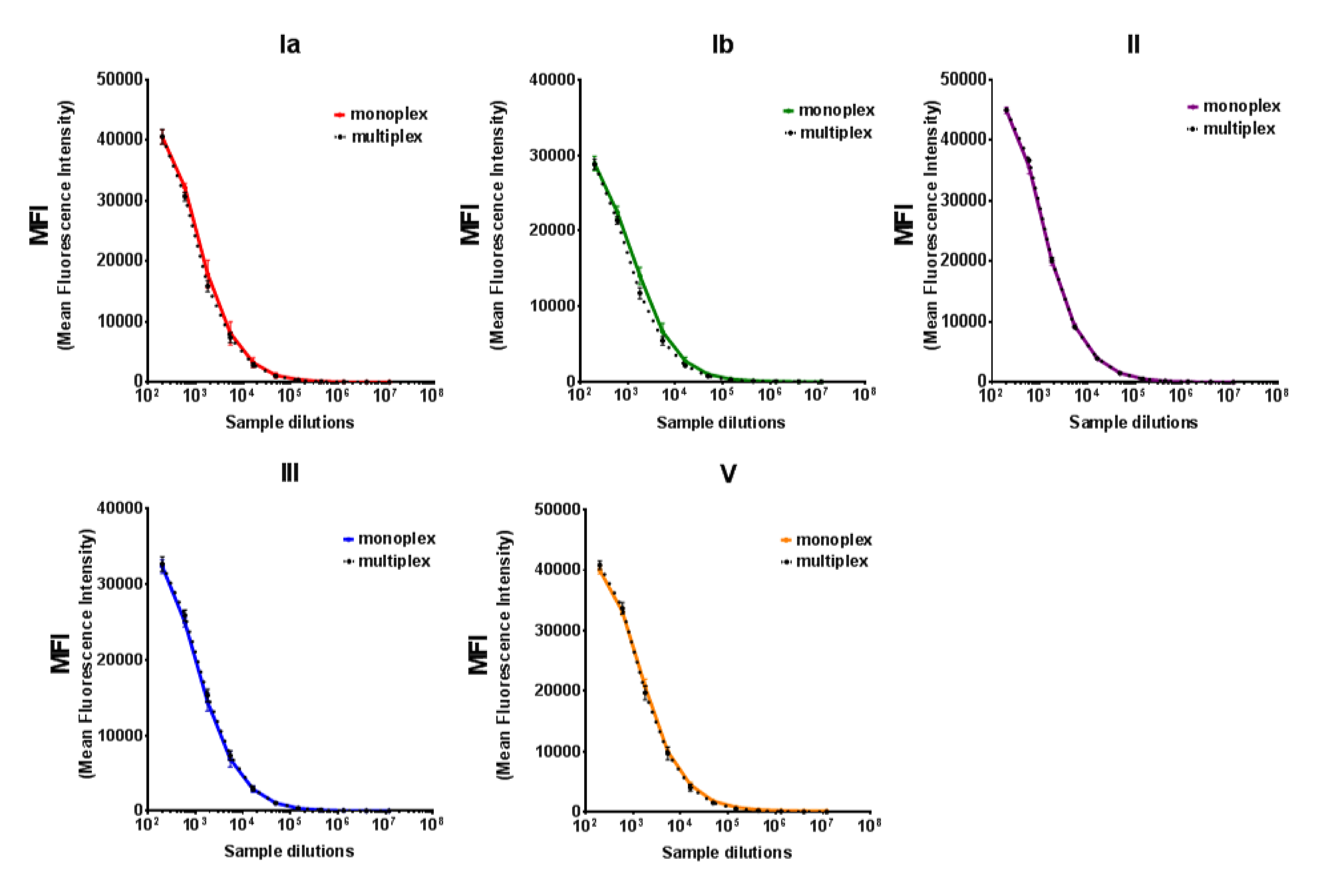

Supplement: FIG S2 [file mSphere.00273-19-sf002.tif]
