# Supplementary material for: Novel Multiplex Immunoassays for Quantification of IgG against Group B Streptococcus Capsular Polysaccharides in Human Sera
Source: mSphere. 2019 Aug 7;4(4):e00273-19. doi: 10.1128/mSphere.00273-19 (PMC6686225; doi:10.1128/mSphere.00273-19)
Supplement: TABLE S2 [file mSphere.00273-19-st002.docx]

| Sample | Biotin-CPS MIA | Sandwich MIA |
| --- | --- | --- |
| SAMPLE_4 | 1,224 | 1,361 |
| SAMPLE_6 | 996 | 1,061 |
| SAMPLE_17 | 490 | 270 |
| SAMPLE_22 | 267 | 228 |
| SAMPLE_36 | 284 | 187 |
| SAMPLE_38 | 538 | 532 |
| SAMPLE_43 | 3,191 | 1,584 |
| SAMPLE_58 | 373 | 405 |
| SAMPLE_66 | 1,422 | 1,375 |
| SAMPLE_75 | 302 | 394 |
