# Supplementary material for: Novel Multiplex Immunoassays for Quantification of IgG against Group B Streptococcus Capsular Polysaccharides in Human Sera
Source: mSphere. 2019 Aug 7;4(4):e00273-19. doi: 10.1128/mSphere.00273-19 (PMC6686225; doi:10.1128/mSphere.00273-19)
Supplement: TEXT S1 [file mSphere.00273-19-s0001.docx]

*Evaluation of the biotinylation percentage:* This value was calculated by determining the total biotin content of the conjugate sample by colorimetric assay using the QuantTag Biotin Quantification Kit (Vector Labs, cod. BDK-2000). Biocytin (Sigma-Aldrich) was used as standard (range 1–10 nmol/ml). The percentage of biotinylation was calculated using the following formula:

$$\% biotin=\frac{nmol/mL biotin}{nmol/mL RU saccharide}\times100$$

*Quantification of the percentage of unreacted capsular polysaccharides (CPSs):* The amount of unreacted polysaccharide was quantified using reverse phase high pressure liquid chromatography, by taking advantage of the strong interaction between streptavidin and biotin. Briefly, two analytical samples were prepared in the mobile phase to a final concentration of 350 µg/mL in biotinylated CPS. Streptavidin (Thermo Fisher), diluted at 5 mg/mL in phosphate buffered saline, was added to one of these samples, in a molar ratio sufficient to completely bind the biotin conjugated to the CPS. A standard curve, in the saccharide concentration range 0.005–0.500 mg/mL, is generated by diluting the unconjugated CPS of the same serotype of the conjugate to be analyzed. The biotinylated and unreacted CPS in the untreated sample will not bind to the column, so they are eluted as a single peak in the flow through following the injection and were used for the total polysaccharide quantification. The biotinylated CPS complexed with the streptavidin will bind to the column and will be retained, hence the peak that elutes following the injection of the streptavidin treated sample will represent the amount of unreacted CPS. Between each set of untreated and treated samples, a blank, a 10 µg/ml biocytin injection, and again a blank were run to remove residual streptavidin from the column. The percentage of unreacted CPS can be calculated using the following formula:

$$\% unreacted CPS=\frac{unreacted CPS (mg/mL)}{total CPS (mg/mL)}\times100$$

The chromatography was done using an Ultimate 3000 (Thermo Fisher Scientific) equipped with a column Aeris C4 200Å 3.6 µm 4.6x250 mm with a C4 guard cartridge (Phenomenex), the detection was performed by UV at wavelength 214 nm and the elution with a mobile phase 10 mM phosphate buffer pH 7.2–7.5 (Carlo Erba) added with 10 mM sodium chloride (Merck) and 5–10% acetonitrile HPLC grade (Sigma-Aldrich), depending on the CPS serotype, followed by a washing step gradient in water:acetonitrile and reconditioning. The data were managed using Chromeleon software, version 6.8 (Thermo Fisher Scientific).

*Structure identity and conformity of the biotin-CPSs:* ^1^H-NMR experiments were recorded on a Bruker Avance III 400-MHz spectrometer equipped with a high precision temperature controller (± 0.1°C) and using a 5-mm broadband probe (Bruker). TopSpin version 3.2 software (Bruker) was used for data acquisition and processing. The NMR analytical samples of biotinylated CPS were prepared by solubilizing ~1.5 mg of dried saccharide, previously obtained by solvent evaporation under vacuum, in 0.75 mL of deuterium oxide (99.9 atom % deuterium; Aldrich) in a glass or plastic vial (i.e. Wheaton or Eppendorf vials). The solutions were mixed to obtain a uniform concentration and subsequently transferred using a pipette to a 5-mm NMR tube (Wilmad or similar). ^1^H-NMR spectra were collected at 25 ± 0.1°C with 16,000 or 32,000 data points over a 10-ppm spectral width and accumulating scans to obtain a signal/noise ratio (S/N) for appropriate peak integration (≥5 S/N or better; typically, 128 scans were recorded) and applying a total recycle time (i.e., five-fold longitudinal relaxation time T_1_ to ensure a full recovery of each signal and to obtain spectrum in quantitative manner). The spectra were weighted with line broadening (generally 0.1–0.2 Hz) and Fourier-transformed. The transmitter was set at the water frequency, which was used as the reference signal (4.79 ppm).
