# Supplementary material for: Novel Multiplex Immunoassays for Quantification of IgG against Group B Streptococcus Capsular Polysaccharides in Human Sera
Source: mSphere. 2019 Aug 7;4(4):e00273-19. doi: 10.1128/mSphere.00273-19 (PMC6686225; doi:10.1128/mSphere.00273-19)
Supplement: TEXT S2 [file mSphere.00273-19-s0002.docx]

*Negative samples.* Negative samples to calculate the limits of blank (LOBs) were selected among the 77 samples analyzed for the comparability study. They presented mean fluorescence intensity values lower than the lower limit of standard curve accuracy (LLSCA) for all dilution points or less than three dilution points with estimated concentrations above a pre-established cut-off. The cut-off was assigned for each serotype considering the IgG concentration corresponding to the LLSCA in the third serial dilution point (1/1,800) and therefore as the LLSCA concentration *1,800. This cut-off was selected because valid determinations require at least three dilution points above the LLSCA. Results below the LLSCA were assigned a concentration corresponding to the LLSCA concentration multiplied by 600 (the second dilution, corresponding to at least two determinations). The LOB was calculated as the value of the [n x 0.95 + 0.5]^th^ ordered observation, n being the total number of measurements.
